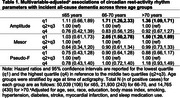# Age‐dependence of the link between circadian rest‐activity rhythm parameters and incident dementia

**DOI:** 10.1002/alz70860_107292

**Published:** 2025-12-23

**Authors:** Sasha Milton, Jiahe Wei, Haizhen Chen, Kristine Yaffe, Yi Fang, Xiao Tan, Yue Leng

**Affiliations:** ^1^ University of California, San Francisco, San Francisco, CA, USA; ^2^ Zhejiang University, Hangzhou, Zhejiang, China; ^3^ San Francisco Veterans Affairs Health Care System, San Francisco, CA, USA; ^4^ Uppsala University, Uppsala, Uppsala, Sweden

## Abstract

**Background:**

Mounting research has revealed a link between circadian differences and dementia risk. However, the directionality of this relationship remains unclear, as most research has focused on older individuals. Examining how this association differs from middle to old age may help clarify the role of circadian shifts as risk factors or prodromal markers of dementia.

**Method:**

We studied 86,734 dementia‐free UK Biobank enrollees (median [range] age=63.3 [42.8‐78.6] years; 43.4% female; 6.5% non‐White). Participants completed seven‐day wrist actigraphy, allowing for the calculation of rest‐activity rhythm (RAR) parameters, including amplitude (rhythm strength), mesor (mean activity level), and pseudo‐F (rhythm robustness). Participants were then followed for all‐cause dementia (ACD), which was determined via International Classification of Diseases codes. We stratified the sample into three age groups (≤65, 66‐70, and >70 years during actigraphy), based on the age distribution. Within each group, we compared incident ACD risk between quartiles of RAR parameters using Cox proportional hazards models. The middle quartiles served as a combined reference category. We tested for interactions between RAR parameters and age.

**Result:**

In total, 779 (0.9%) individuals developed ACD during a median (interquartile range) follow‐up time of 7.9 (7.3‐8.4) years, with 106 (0.21%), 243 (1.1%), and 430 (2.9%) cases in the ≤65, 66‐70, and >70 groups, respectively. After adjustment for age, sex, race, education, body mass index, smoking, hypertension, diabetes, stroke, myocardial infarction, and sleep medication use, participants in the lowest quartile of amplitude or mesor exhibited increased ACD risk relative to individuals in the middle two quartiles, but only in the 66‐70 group (hazard ratio, HR [95% confidence interval, CI] for amplitude: 1.71 [1.26,2.33]; mesor: 2.05 [1.50,2.78]) and the >70 group (HR [95% CI] for amplitude: 1.36 [1.09,1.71]; mesor: 1.50 [1.20,1.89]). The highest quartiles of amplitude and mesor were not associated with ACD risk, nor was pseudo‐F. RAR‐age interactions were not significant (*p* >0.05).

**Conclusion:**

Low RAR amplitude and mesor were associated with an elevated risk of incident ACD in adults aged above 65 years, highlighting circadian disruption as a potential prodromal marker of dementia in older adults.